# Draft Genome Sequences of Three Newly Identified Species in the Genus *Cronobacter*, *C. helveticus* LMG23732^T^, *C. pulveris* LMG24059, and *C*. *zurichensis* LMG23730^T^

**DOI:** 10.1128/genomeA.00783-13

**Published:** 2013-09-26

**Authors:** Naqash Masood, Karen Moore, Audrey Farbos, Sumyya Hariri, Konrad Paszkiewicz, Ben Dickins, Alan McNally, Stephen Forsythe

**Affiliations:** Pathogen Research Group, School of Science and Technology, Nottingham Trent University, Nottingham, United Kingdoma; Wellcome Trust Biomedical Informatics Hub, Biosciences, University of Exeter, Exeter, United Kingdomb

## Abstract

*Cronobacter helveticus*, *Cronobacter pulveris*, and *Cronobacter zurichensis* are newly described species in the *Cronobacter* genus, which is associated with serious infections of neonates. This is the first report of draft genome sequences for these species.

## GENOME ANNOUNCEMENT

The *Cronobacter* genus until recently consisted of seven species, *C. sakazakii*, *C. malonaticus*, *C. turicensis*, *C. universalis*, *C. muytjensii*, *C. dublinensis*, and *C. condimenti* (1). In 2013, three new *Cronobacter* species were proposed, *C. zurichensis*, *C. pulveris*, and *C. helveticus* (2). Due to the association of *Cronobacter* with fatal neonatal infections there is an international requirement for powdered infant formula to be microbiologically tested for all members of the *Cronobacter* genus (Codex Alimentarius Commission, code of hygienic practice for powdered formulae for infants and young children, http://www.codexalimentarius.net/download/standards/11026/CXP_066e.pdf). Therefore, the genome sequencing of these newly described species is warranted for better understanding of the genus diversity and improved detection methodology. This was undertaken using *C. helveticus* strain LMG23732^T^, *C. zurichensis* strain LMG23730^T^, and *C. pulveris* strain LMG24059 isolated from powdered infant formula.

Bacterial DNA was extracted from 1-day cultures using a GenElute bacterial genome kit (Sigma-Aldrich) and sequenced using an Illumina HiSeq 2500 sequencing system. *De novo* assembly was performed using Velvet (3). For further annotation we used the SEED-based automated annotation system provided by the RAST server (4).

*C. helveticus* LMG23732^T^, *C. pulveris* LMG24059, and *C. zurichensis* LMG23730^T^ generated 8,351,512, 5,000,582, and 6,613,368 high-quality paired-end reads of 150 bp in length, respectively. The genome sizes were 4,530,369 bp, 4,900,556 bp, and 4,246,797 bp with G+C contents of 56%, 56.6%, and 57.8%, respectively. These were in 79, 125, and 103 contigs with 4,315, 4,630, and 3,883 coding sequences (CDS), respectively, and 30-fold coverage. The CDS include traits previously described in *Cronobacter* (5). These included genes associated with iron acquisition, stress response, heavy metal resistance (arsenic, copper cobalt, zinc and cadmium), and phages. Genes encoding proteins associated with several virulence-associated traits such as adhesion and antibiotic resistance, including fluoroquinolones, fosfomycin, β-lactamases, and multidrug efflux pumps, were also determined.

### Nucleotide sequence accession numbers.

The genome sequences of *C. helveticus*, *C. pulveris*, and *C. zurichensis* have been deposited in GenBank under the accession numbers AWFX00000000, AWFY00000000, and AWFZ00000000, respectively.
